# The Desire for Multiple Pregnancy among Patients with Infertility and Their Partners

**DOI:** 10.1155/2014/301452

**Published:** 2014-07-22

**Authors:** Ida Lilywaty Md Latar, Nuguelis Razali

**Affiliations:** Department of Obstetrics & Gynaecology, Universiti Malaya, 59100 Kuala Lumpur, Malaysia

## Abstract

*Objective*. To study the predictors for desire for multiple pregnancies and the influence of providing information regarding the maternal and fetal complications associated with multiple pregnancies on their preference for multiple pregnancies.* Methods*. Couples attending an infertility clinic were offered to fill up a questionnaire separately. Following this, they were handed a pamphlet with information regarding the risks associated with multiple pregnancies. The patients will then be required to answer the question on the number of pregnancies desired again.* Results*. Two hundred fifty three out of 300 respondents completed the questionnaires adequately. A higher proportion of respondents, 60.3% of females and 57.9% of males, prefer singleton pregnancy. Patients who are younger than 35 years, with preexisting knowledge of risks associated with multiple pregnancies and previous treatment for infertility, have decreased desire for multiple pregnancies. However, for patients who are older than 35, with longer duration of infertility, and those patients who have preexisting knowledge of the increased risk, providing further information regarding the risks did not change their initial preferences.* Conclusion*. Providing and reinforcing knowledge on the risks to mother and fetus associated with multiple pregnancies did not decrease the preference for multiple pregnancies in patients.

## 1. Introduction

Assisted reproductive techniques (ART) have enabled many childless couples to achieve their dream of having a child of their own. The number of women undergoing ART treatment has increased tremendously over the past three decades, leading to more than 5 million children conceived by this treatment [1]. However, as ART traditionally involved ovarian stimulation and replacement of more than one embryo, it had contributed to a significantly higher number of multiple births.

The multiple birth rate from 376,971 European IVF treatment cycles in 2007 was reported as 22.3% (21.3% twins and 1% triplets), similar to rates in 2005 and 2006 (21.8 and 20.8%, resp.) [2]. The data from the Society for Assisted Reproduction Technology (SART) registry in the USA, based on 108,130 ART cycles, revealed a multiple birth rate of 35.4, of which 31.8% were twins, 3.5% were triplets, and 0.1% were higher order multiple [3].

As compared to singleton, twin and higher order pregnancies have contributed significantly to preterm deliveries. Prematurity, which is a main cause of neonatal morbidity and mortality, occurs in nearly one-half of all multiple pregnancies. It was reported that 42% of twins are born before 37 completed weeks, compared to 8% of singletons [4, 5]. The risk of mortality is increased six-fold in twins and 10–20-fold in triplets compared to singletons [6]. Multiple pregnancies are also associated with higher rate of spontaneous abortion and intrauterine fetal demise. It is also more likely to result in neonatal and infant death, cerebral palsy, and congenital birth defects [7].

Maternal complications that may be associated with multiple pregnancies are hypertensive disorders, abruptio placentae, caesarean delivery, and postpartum haemorrhage [8]. Women who had multiple births are at increased risk for depression and experiencing higher parenting stress [9].

Furthermore, the economic effect on society and the economic and emotional stresses on families that are associated with raising twins, triplets, and more children are becoming increasingly apparent [8, 10]. Following these concerns, multiple pregnancies are considered as a serious complication of assisted reproductive techniques (ART) and the American Society for Reproductive Medicine has actually designated reduction in the incidence of multifetal pregnancy resulting from ART “an essential goal for ART programs and their patients” [11].

Single embryo transfer (SET) is increasingly practiced worldwide in an effort to reduce rates of multiple births associated with ART. The rate of single embryo transfer was about 20% in Europe in 2005, but much higher rates are reported in some countries (69% in Sweden in 2005 and 57% in Australia and New Zealand in 2006) [12]. Although SET prevents multiple pregnancies, there are concerns that it might reduce pregnancy rates. The data from the Cochrane database 2014 showed that a live births rate (LBR) following a single cycle of double embryo transfer was 45% while the LBR following a single cycle of single embryo transfer was between 24% and 33%. The LBR following repeated single embryo transfer would be between 31% and 44%. However, the risk of twins was about seven times higher after double embryo transfer [13]. In Sweden, SET represented 67% of all transfers in 2004, with an almost unchanged delivery rate of 27% per transfer, whereas the IVF multiple birth rate was reduced to 5.6% [14]. The Turkish government implemented a new legislation starting March 2010 in an attempt to promote single-embryo transfer. According to the new regulation, only one embryo transfer is permitted in the first and second treatment cycles of patients under 35, a maximum of two embryos can be transferred in the third or further cycles, and a maximum of two embryos can be transferred to patients aged above 35 years [15].

A study by Kutlu et al. following the implementation of the new legislation showed that the overall clinical pregnancy rates were decreased from 39.9% to 34.5% and multiple pregnancy rates were decreased from 23.1% to 5.3%. The difference in pregnancy rates was not statistically significant while the difference in multiple pregnancy rates was statistically highly significant [16].

Regardless of the concerns above, a large proportion of infertility patients still appear to favour multiple pregnancies as an ideal treatment outcome [17]. For infertile patients who want more than one child, twin deliveries represent a favourable and cost effective treatment outcome that should be encouraged, in contrast to the current medical consensus [18, 19]. These preferences have been strongly associated with the patients' lack of knowledge about fertility treatment and multiple pregnancy as well as the associated risks [17, 20]. Previous data has shown that simple educational materials can improve knowledge of twin pregnancy risks and affect decision making [21]. In developing practices and policies of ARTs, it is important to take into account the interest of all parties involved. At a minimum, providers and patients need to be educated about the risks of multiple gestations so that steps can be taken to prevent adverse outcomes [14].

In Malaysia, very few data has been published to evaluate these matters. As ART in Malaysia is considered costly and not financially supported by the government, it would be beneficial to assess the patients' knowledge and preferences on this particular ground to further assist the clinician in fertility treatment and management of the patients.

The aim of this study is to determine the preferences on the number of pregnancies among infertility patients and their partners and to assess the effect of providing or reinforcing the information regarding the risks associated with multiple births. These include their demographic picture, their desire towards multiple pregnancies, and their knowledge about its risks as well as other factors that may affect their preferences such as age, sex, financial level, and prior knowledge on the risks of multiple pregnancies.

## 2. Materials and Methods

All the women and their spouses attending the infertility clinic in University Malaya Medical Centre (UMMC), Kuala Lumpur, during the period of six months from December 2009 until June 2010 were offered to fill in a pre-prepared questionnaire comprising 3 sections (Appendix A). The questionnaire was prepared in 2 languages (Bahasa Malaysia and English) as preferred by the subjects recruited. The patients and their partners were required to self-complete the questionnaire separately without consulting each other. The subjects include those first time attendees as well as patients coming for follow-up visits. The patients were required to respond to the questionnaire only once throughout their course of treatment in the infertility unit. The research was reviewed and approved by the UMMC Research Ethical Board (IRB reference number: 782.3).

The first section of the questionnaire includes subjects' demographic data such as age, sex, ethnicity, level of education, occupation, and estimated total family income. The next section of the questionnaire includes data on their fertility as well as any prior treatment received. At the end of the second section, subjects will be asked in order of preferences regarding the number of pregnancies they desire. This was prepared in a 4-point scale fashion with 1 indicating the most desirable number of pregnancies and 4 indicating the least. Following this, subjects will be given a pre-prepared multiple languages pamphlet that will provide information on the association of multiple pregnancies with fertility treatment as well as its risks (Appendix B). Subjects will then be required to answer the question on the number of pregnancy desired that was previously asked again.

Our analysis focused on the patients and their partners' desire for multiple pregnancies, their knowledge of the risks associated with it, and the association between demographic, infertility and treatment history, and knowledge and its effects on their desire for multiple pregnancies. Data was analysed using multiple logistic regression analysis (SPSS version 17). We identified independent variable associated with positive response as desire for multiple pregnancies. Continuous variables were analysed by means of 2-sample* t*-test and categorical responses were analysed by Fisher exact or chi-square test.

## 3. Results

A total of 300 questionnaires were distributed throughout the study. Out of these, 253 respondents completed the questionnaire while the remaining questionnaires were not included in the analysis as they were not fully completed with omitted answers. The 253 respondents included 107 men and 146 women.

Demographic data with the baseline characteristics of the respondents are shown in Table 1. The mean age of the female and male respondents was 31.7 years and 33.5 years, respectively. The mean duration of infertility for both groups was 51 months for female and 42.5 months for male respondents. Both groups have the mean total monthly income of around RM3500. Most of our respondents were well educated with almost 50% of the female and male respondents having at least a diploma or a degree in their respective fields. A smaller proportion of the female respondents already had children prior to this treatment as compared to their male partners (9.3% versus 14.4%). As expected, more women had received previous treatment as compared to their male counterparts (52.1% versus 33.6%).

More female and male respondents desired singleton pregnancy rather than multiple pregnancies but the difference between the two genders was not significant (*P* = 0.71) (Table 1). The possible associated factors were cross-tabulated according to desired number of foetuses in Table 2. A significant number of younger patients (<35 years old taken as the baseline) actually preferred singleton pregnancy compared to the older age group. Similarly, preexisting knowledge of risks of multiple pregnancies also significantly influenced the desire for singleton pregnancy. Total income and previous fertility treatment did not affect desire for multiple or singleton pregnancy.

Among those who desired twins and higher orders pregnancies, 46.7% of male and 44.8% of female respondents gave advancing age as the reason for preferences. 11.1% of male and 15.5% of female respondents, respectively, stated long duration of trying to conceive as the reason of preferring twins or higher orders pregnancies. Other reasons given were high cost of fertility treatment (2.2% versus 5.1%), wanting to complete family faster, worry of inability, or difficulties to get pregnant again or health factor.

Univariate and multivariate analysis was carried out to look for predictors of preference of multiple pregnancies (Table 3). Age more than 35 years is associated with increased preference while previous fertility treatment and preexisting knowledge on the risks of multiple pregnancies was associated with reduced preference.

Out of those who desired multiple pregnancies prior to exposure to knowledge of its risks, only 24.1% of female respondents and 37.8% of male respondents changed their preference from multiple pregnancies to singleton after being exposed to the information about multiple pregnancies. Univariate and multivariate analysis was performed to identify factors that are associated with continuous desire for multiple pregnancy even after given information regarding the risks of multiple pregnancies (Table 4). Respondents who are more than 35 years old and those with preexisting recognition regarding the risks of multiple pregnancy were significantly more likely to continue with their preference even after given additional information.

## 4. Discussion

Contrary to the previous data, more patients in our setting considered singleton pregnancy as an ideal treatment outcome even before being exposed to the knowledge of multiple pregnancies itself [19, 22]. Initially, we hypothesized that more patients would desire multiple pregnancies as ART is not funded in our country; therefore, multiple pregnancy is deemed as more cost effective for them. This positive finding might be because of the background of our patients who are mostly well educated with more exposure to the information itself even before being informed to them and perhaps cost really is not an issue. This finding is similar to other studies [23].

Our study has found that there is no significant difference in the desire of multiple pregnancies among our male and female respondents. This is in accordance with the previous data available which has shown that patient's sex did not affect desire for multiple births [22, 24].

Another interesting finding is that those patients who are younger have been found to have a significant desire for singleton as compared to multiple pregnancies as the ideal treatment outcome. This is in contrast to the study by Ryan et al. which showed that younger women preferred multiple pregnancies [17]. A possible explanation for this may be that younger patients are not pressured to complete their family earlier as compared to the older patients. However, on the other hand, there was no clear evidence to show significant preferences towards multiple pregnancies among older age groups.

Patients who are aware of the increased fetal risks associated with multiple pregnancies were significantly less likely to desire this outcome. This suggests that patients' prior information and knowledge regarding the risks will reduce their desire of having multiple pregnancies as the treatment outcome.

After the patients have been given the information regarding the risks of multiple pregnancies following fertility treatment, only a small proportion of those who had first chosen multiple pregnancies changed their decision to singleton pregnancy and this finding was not statistically significant. This may be due to the fact that those who opted for multiple pregnancies were already aware of the risks but still prefer multiple pregnancies due to reasons such as increasing age or desire to complete family faster. Other reasons such as desire for siblings, a positive attitude towards twins, and a wish to minimize physical and psychological stress through having as few IVF treatments as possible were given in a Danish study [25].

Various methods have been employed in order to provide patients with information regarding the risks associated with multiple pregnancies. This includes provision of information leaflets, additional discussion sessions, risk perception survey, and communication strategies utilizing the framing effect and fear appeal [21, 26, 27]. An empowerment programme which consisted of a decision aid kit, support of a nurse, and reimbursement of an additional treatment cycle was developed by van Perperstraten et al. in an attempt to persuade patients to choose elective SET. Their study reported that patients who were administered the empowerment strategy were more likely to choose elective SET, but the differences between the empowerment and control group was much lower than the estimated goal of 25% based upon power calculations [28]. The results for these studies have been conflicting. Thus, the value of providing information regarding risks associated with multiple births to infertile patients and empowerment program with reimbursement has been shown to be limited [28]. 

Although more of our patients preferred singleton pregnancy compared to multiple pregnancies during the survey, we do acknowledge the fact that this might not translate to increase preference of single embryo transfer (SET) over double embryo transfer (DET). Højgaard et al. noted that the majority of their infertile couples currently in treatment preferred twins (58.7%) to one child at a time (37.9%) but a larger majority (78.5%) planned to have two embryos transferred in the next treatment. There was no association between opting for twins and having received information and feeling well informed [25]. The preference for DET is not only explained by a wish to have a high success rate and, thus, avoiding more treatments but also reflects a deliberate wish to have twins in the majority of couples [25]. In a review of twenty papers by Leese and Denton, patients in most studies would rather choose double-embryo transfer than single, mainly to maximize their chances of achieving a pregnancy and did not necessarily reflect a preference for twins [29]. Similarly, Newton et al. also noted both women and men in their study tended to view 2ET as the most desirable option. However, attitudes toward SET changed markedly after providing patients with information about the risks of twin pregnancy [23].

The patients' preference for a singleton pregnancy as the preferred treatment outcome may indirectly assist the decision making process for the clinician in treating them. Based on our finding, with adequate counselling, younger patients might be more accepting of SET. Apart from providing risk information, patients also should be informed regarding the success rate of SET. To further improve the acceptability of SET, the outcome with cryopreserved embryos and protocols for frozen embryo transfer also need to be improved. It is worthwhile to note that even though most of our patients preferred singleton pregnancy, there is still a sizeable proportion of patients who preferred multiple pregnancy and are willing to take the risks associated with it. Thus, clinicians may need to take this preference into account after appropriate counselling and the absence of any medical conditions.

## 5. Conclusion

A higher proportion of patients with infertility actually prefer singleton pregnancy compared to multiple pregnancy. The desire on multiple pregnancies among patients with infertility is not affected by sex or financial factors. Younger patients significantly prefer singleton as compared to multiple pregnancies. Patients with prior knowledge on the risks of multiple pregnancies were significantly less likely to desire this outcome. However, for those patients who prefer multiple pregnancies, reinforcing the knowledge on risks of multiple pregnancies did not reduce their desire.

## Limitations

The limitation of this study was the pamphlet of information given to the patients. Different respondents may interpret the magnitude of the risks implied by the pamphlets differently.

## Figures and Tables

**Table 1:** Demographic data and baseline characteristics of respondents.

	Female (*n* = 146)	Male (*n* = 107)	*P* value
Age (years), mean (SD)	31.7 (4.4)	33.5 (5.5)	*P* 0.004

Duration of infertility (months), mean (SD)	51.0 (34.8)	42.5 (26.4)	*P* 0.29

Total income (RM), mean (SD)	3574.7 (1479.1)	3532.7 (1401.7)	*P* 0.82

Level of education, *n* (%)			
Primary school	2 (1.4%)	4 (3.7%)	
Secondary school	76 (52.1%)	57 (53.3%)	
Diploma/degree	63 (43.2%)	43 (40.2%)	
Postgraduate	5 (3.4%)	3 (2.8%)	

History of previous children, *n* (%)	10 (9.3%)	21 (14.4%)	

Previous treatment received, *n* (%)	76 (52.1%)	36 (33.6%)	

Desired number of babies with next fertility treatment, *n* (%)			
Singleton	88 (60.3%)	62 (57.9%)	*P* 0.71
Multiple pregnancies	58 (39.7%)	45 (42.1%)	

Decision changed following information regarding multiple pregnancies, *n* (%)	14 (24.1%)	17 (37.8%)	*P* 0.13

**Table 2:** Desire in order of pregnancy of 253 infertility patients and their partners with associated information.

	Desired singleton (*n* = 150)	Desired multiple pregnancies (*n* = 103)	*P* value
Total income > RM 3500	78 (52.0%)	54 (52.4%)	*P* 0.95

Age < 35 years old	121 (80.7%)	65 (63.1%)	*P* 0.02
Age > 35 years old	29 (19.3%)	38 (26.9%)	

Previous treatment received	63 (42.0%)	49 (47.6%)	*P* 0.38

Preexisting recognition of risks of multiple pregnancies	121 (80.7%)	66 (64.1%)	*P* 0.002

**Table 3:** Univariate and multivariate analysis of predictors for multiple pregnancies outcome.

	Number of patients	Univariate analysis	*P*	Multivariate analysis	*P*
		Hazard ratio		Hazard ratio	
		(95% CI)		(95% CI)	
Total	253				

Age < 35 years	186	1.00		1.00	
Age ≥ 35 years	67	2.44 (138–4.31)	0.002	2.29 (1.26–4.16)	0.014

Treatment IUI					
No	230	1.00			
Yes	23	0.41 (0.17–0.98)	0.044		

Preexisting recognition of risks of multiple pregnancy					
No	54	1.00		1.00	
Yes	187	0.30 (0.16–0.56)	<0.001	0.30 (0.16–0.57)	<0.001

**Table 4:** Univariate and multivariate analysis of predictors for a continuous desire of multiple pregnancies outcome.

	Number of patients	Univariate analysis	*P*	Multivariate analysis	*P*
		Hazard ratio		Hazard ratio	
		(95% CI)		(95% CI)	
Total	103				

Age < 35 years	65	1.00		1.00	
Age ≥ 35 years	38	3.33 (1.22–9.11)	0.019	3.57 (1.24–10.43)	0.019

Duration of infertility					
<5 year	70	1.00			
≥5 years	33	3.31 (1.14–9.63)	0.028		

Preexisting recognition of risks of multiple pregnancy					
No	35	1.00	0.001	1.00	
Yes	66	4.32 (1.76–10.60)		4.59 (1.79–11.72)	0.001

## Appendices

### A. The Desire for Multiple Pregnancies Among Patients with Infertility and Their Partners

#### A.1. Questionnaire

1. Age: ………………years old
2. Sex: ………………Male/Female
3. Race: ………………Malay/Chinese/Indian/Others
4. Level of education: Primary school/Secondary school/Degree/Postgraduate
5. Occupation (please state): ………………
6. Clinic attendance: First visit/Follow up
7. How long have you and your partner have been trying to conceive? …….. years and ………months
8. Number of children you had before (including with previous partners, if any?) ………………
9. Which of the following fertility treatments have you had before? (Please tick correct answer)
10. …………… Fertility pills
11. ………….. Surgery to open /repair blocked tubes
12. ………… IUI (Stimulation of ovaries & insemination of sperm into uterus)
13. ………… IVF (In Vitro Fertilization/test tube baby treatment)
14. ………… others.
15. Have you ever been counseled before regarding the risk of multiple pregnancies following fertility treatment? YES/NO.
16. If the current fertility treatment is successful, what would be the preferred number of babies that you would like to have (please put in order of numbers, 1 for most desirable, followed by 2, 3 and 4).
17. …….. Singleton pregnancy (1 baby)
18. …….. Twins (2 babies)
19. …….. Triplets (3 babies)
20. …….. Quadruplets (4 babies)

Please state your reasons for making the choices above: ……..……..……..……..

(12) Do you think that there are more dangers for the babies in a multiple pregnancy (2 or more babies) compared to singleton (1 baby)? YES/NO.

#### A.2. Please Answer This Question Again after You Have Read the Information Leaflet Provided (Your Answer May Be Different from the Previous Answer that You Have Given)

(11) If the current fertility treatment is successful, what would be the preferred number of babies that you would like to have (please put in order of numbers, 1 for most desirable, followed by 2, 3 and 4)
……….. Singleton pregnancy (1 baby)
……….. Twins (2 babies)
……….. Triplets (3 babies)
……….. Quadruplets (4 babies)

### B. Information Leaflet

Dear participants,

Please read through this information leaflet before answering the last question:

Fertility treatments are associated with increased number of multiple pregnancies
Multiple pregnancies have generally higher risks as compared to single (one baby) pregnancy. The risks involve the mother as well as the fetus/baby.

*The mother is at increased risk of several pregnancy complications, including*:

1. Miscarriage
2. Hyperemesis (Severe vomiting in early pregnancy)
3. Premature labour and delivery
4. Anaemia (reduced red blood cells)
5. Pre-eclampsia (complicated hypertension in pregnancy)
6. Antepartum hemorrhage (vaginal bleeding during pregnancy)
7. Postpartum hemorrhage (excessive vaginal bleeding after delivery)
8. Polyhydramnios (Excessive amniotic fluid) which may lead to abdominal discomfort and breathlessness
9. Operative delivery (caesarean section)
10. Prolonged stay in hospital

*The babies are at increased risk of*:

(1) Preterm labour: Twins are 5 times more likely to be born preterm compared to singleton. Almost 15% of triplets (pregnancies with 3 babies) deliver before 30 weeks of gestation. Preterm babies have higher risks of:

(i) Neurological problems like encephalopathy, cerebral palsy and intraventricular hemorrhage
(ii) (Bleeding in brain)
(iii) Retinopathy (poor development of the retina of the eye)
(iv) Developmental disability
(v) Heart problems
(vi) Respiratory problems: Respiratory distress syndrome, chronic lung disease
(vii) Gastrointestinal and metabolic issues can arise from low sugar level, feeding difficulties, low calcium, inguinal hernia and necrotizing enterocolitis (immature gut)
(viii) Hematologic complications include anemia, low platelet and jaundice.
(ix) Severe infection including sepsis, pneumonia and urinary tract infection.
(1) Intrauterine growth restriction (babies born below the normal weight range): 25-33% risks
(2) Single fetal death
(3) Cerebral Palsy
(4) Abnormal babies
(5) Twin to twin transfusion syndrome (TTTS): Complication of unequal blood supply when two or more fetuses share a single sac and placenta. Severe TTTS has a 60-100% fetal death rate.
